# Tesserae on Venus may preserve evidence of fluvial erosion

**DOI:** 10.1038/s41467-020-19336-1

**Published:** 2020-11-13

**Authors:** S. Khawja, R. E. Ernst, C. Samson, P. K. Byrne, R. C. Ghail, L. M. MacLellan

**Affiliations:** 1 Department of Earth Sciences, Carleton University, 1125 Colonel By Drive, Ottawa, Ontario K1S 5B6 Canada; 2 Faculty of Geology and Geography, Tomsk State University, 36 Lenin Avenue, Tomsk, 634050 Russia; 3 Department of Marine, Earth, and Atmospheric Sciences, North Carolina State University, Raleigh, NC 27695 USA; 4 Department of Earth Sciences, Royal Holloway, University of London, Egham, TW20 0EX UK

**Keywords:** Planetary science, Astronomy and planetary science

## Abstract

Fluvial erosion is usually assumed to be absent on Venus, precluded by a high surface temperature of ~450 °C and supported by extensive uneroded volcanic flows. However, recent global circulation models suggest the possibility of Earth-like climatic conditions on Venus for much of its earlier history, prior to catastrophic runaway greenhouse warming. We observe that the stratigraphically oldest, geologically most complex units, tesserae, exhibit valley patterns morphologically similar to the patterns resulting from fluvial erosion on Earth. Given poor topographic resolution, we use an indirect technique to recognize valleys, based on the pattern of lava flooding of tesserae margins by adjacent plains volcanism. These observed valley patterns are attributed to primary geology, tectonic deformation, followed by fluvial erosion (and lesser wind erosion). This proposed fluvial erosion in tesserae provides support for climate models for a cool, wet climate on early Venus and could be an attractive research theme for future Venus missions.

## Abstract (web summary)

The authors here use Magellan data to interpret geomorphological features on Venus and present a strong hypothesis for fluvial erosion.

## Introduction

Venus is commonly referred to as Earth’s sister planet because of the proximity of the two in the Solar System and their similarities in size, mass, density, composition, and surface gravity. In contrast to Earth, however, Venus today exhibits a lack of plate tectonics and boasts high surface temperatures (~450 °C) and a dense CO_2_-dominated atmosphere^1^. The most detailed radar image data for Venus were provided by NASA’s 1990–1994 Magellan mission, which collected multiple datasets including high-resolution (~100 m/pixel) images of 98% of the planet’s surface using Synthetic Aperture Radar (SAR) and altimetry data^2^.

**Fig. 1:**
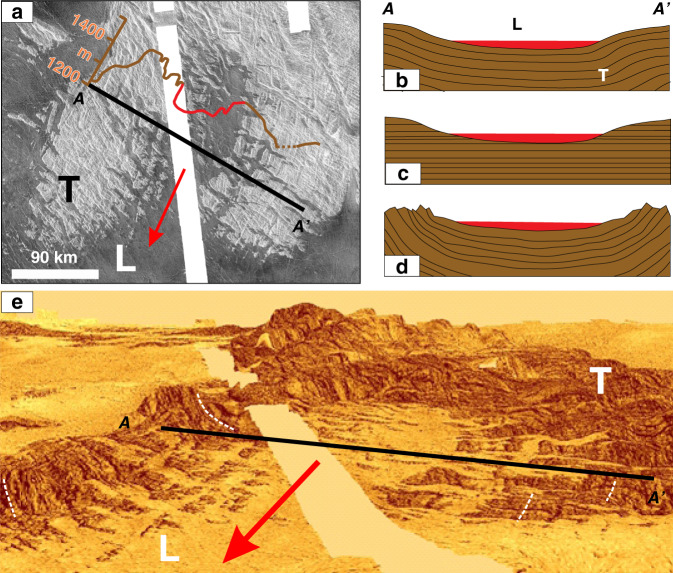
Evidence for erosion in the southern portion of Salus tessera. **a** Left-looking Cycle 1 Magellan SAR image (central coordinates 4.4°S, 48.2°E); north is up, showing tessera (light grey and lineated) with a re-entrant area that has been flooded by younger plains lavas (featureless dark grey). White band marks absence of Magellan SAR data, but topographic data are available in this region. The topographic relief between the valley (covered by lava flows) and bordering tesserae hills is about 100 m (along profile line A–A’). Dotted portion of brown line extrapolates across a topographic data artefact (appearing as a topographic ‘hole’) in the dataset. Red portion of topographic profile corresponds to the portion flooded and embayed by plains lavas. Edge of main flooding (red line) on left side extrapolated from the SAR image on both sides of the data gap. **b**–**d** End-member generalized alternative geological interpretations for cross-section A–A’ of which only the third (**d**) fits the observations: **b** a purely tectonic interpretation whereby these landforms are a central syncline and bordering anticlines; **c** a purely fluvial erosional interpretation, or **d** initial tectonic activity (synclinal folding) followed by fluvial erosion. T = tesserae and L = younger mafic lava flows. **e** Elevated oblique view (looking north) generated by draping the SAR image onto Magellan altimetry topography using the software ArcScene. Vertical exaggeration is 20× and the viewing elevation angle is about 20°. The tessera is orange and the younger mafic lava flows are light yellow, and the smooth yellowish-white band is the missing data. Noteworthy are the white dashed lines in **e** that correspond to prominent lineament sets that may reflect inward dipping strata on both flanks of the inferred syncline.

Tesserae (Fig. 1a, e) are tectonically complex units that occupy ~8% of the surface of Venus, commonly occurring as high-standing crustal plateaus that are embayed by lava flows from the adjacent volcanic plains. Tesserae are inferred from superposition relationships to be the oldest regions on the planet^3–6^. The nature of the pre-deformation terrain, including mode of emplacement, and whether tesserae compositions are mafic or felsic, as well as the causes and timing of deformation, remain unclear^3–6^.

The current high temperatures (450 °C) and pressures (9 MPa) on Venus are generally interpreted to mean that the surface of Venus does not display evidence for extensive erosion^7^, which is convincingly true for stratigraphically younger lobate flows that exhibit simple superposition of flow units and have radar backscatter properties consistent with primary flow morphology and rheology^8,9^. Additional surface features such as contractional structures called wrinkle ridges^10,11^, graben–fissure systems^12,13^, and lava channels (including canali and sinuous rilles)^14–20^ all have significant topographic signatures that can be partially obscured or removed by subsequent erosion. A fluvial rather than volcanic origin for canali has been considered^16,17^, but is generally rejected on the basis of the absence of flowing water due to the high atmospheric temperatures^16,20^. Impact craters in volcanic plains areas are mostly pristine except where affected by younger volcanism^21,22^. These observations imply a lack of fluvial erosion on Venus, consistent with the planet’s current high surface temperature.

Recent modelling, however, suggests a re-consideration of whether flowing water has modified the stratigraphically oldest units, tesserae. A global circulation model for Venus suggests that Earth-like conditions could have existed for most of Venusian history, and that a runaway greenhouse effect changed Venus’ climate catastrophically and led to loss of water^23–26^. The cause of this greenhouse effect is typically linked to CO_2_ degassing during major volcanic eruptions, the timing and duration of which are the subject of debate, ranging from a catastrophic, global volcanic resurfacing event to steady-state resurfacing, yielding mean surface age estimates of ~750 to 150 Ma^21,27,28^. As tesserae predate the widespread mafic plains volcanism linked to this proposed resurfacing, they may have formed prior to runaway greenhouse warming. If these enigmatic surface units do date from before Venus’ climate catastrophe, then a detailed assessment of their morphology might allow evaluation of whether they formed under climatic conditions where liquid water was present on Venus and fluvial erosion could occur.

A clear signal of water erosion would be the recognition of fluvial valley systems. The first hurdle to recognizing tesserae erosion is the uncertainties in Magellan altimetry topographic data, which have vertical and horizontal resolutions of approximately 50–100 m and 10–20 km, respectively^29–31^. Higher-resolution stereo topographic maps produced from combining Cycles 1, 2, and Cycle 3 Magellan SAR data^4,31,32^ offer an improved horizontal resolution of 1–2 km, but such data are only available for only 20% of the planet^31^.

**Fig. 2:**
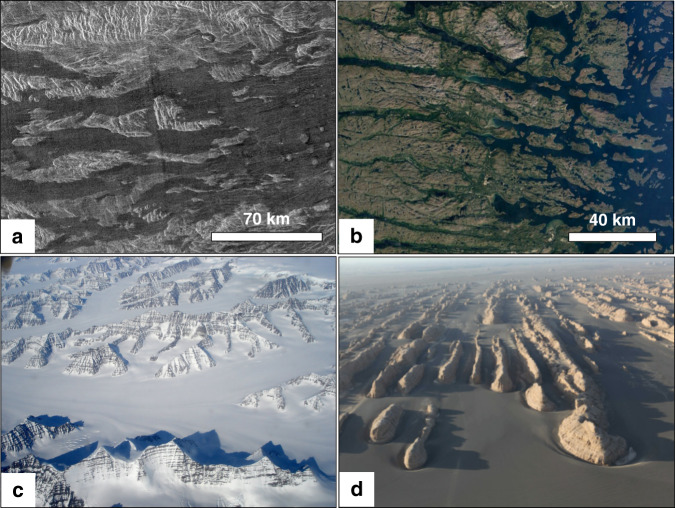
Recognizing topographic highs above a smooth datum. Comparisons of volcanically flooded tessera with topographic highs emerging above a lower, relatively smooth datum on Earth. **a** Magellan left-looking SAR image from Venus at 6.4°S, 62.1°E (central coordinates) showing portions of tesserae (light grey) surrounded by plains units (dark grey). **b** Fjords flooded by water in Nain, Labrador, Canada (Source: Google maps) (56.5°N, 61.7°W). **c** Nunataks (high-standing mountains) surrounded and embayed by a field of ice and snow; image from South of Constable point in East Greenland, courtesy of Carl Skou, 2020 http://www.kuummiut.com/2010/03/glaciersnuataks-and-kuummiut.html. **d** Yardang field bordering the desert of Kumtagh, China (42.7°N, 90.3°E)^52^, with individual features that are hundreds of metres long.

Here we have taken a different and unambiguous proxy approach to identify paleo-valleys within tesserae. We consider only those portions of tesserae, typically their margins, which are partially flooded by younger lava flows fed from the surrounding plains (Fig. 1a). The lava flooding represents an original semi-horizontal surface that readily discriminates areas of higher elevation from paleo-valleys. Even in cases where subsequent local later uplift occurred (affecting both tessera and embayed younger lava flows), the paleo-valley patterns would still be reliably revealed by the flows embaying the tesserae. On Earth, flooded fjords, nunataks, and yardangs are conceptually similar to lava flooding where liquid water is the reference semi-horizontal surface for flooded fiords (Fig. 2b), ice for nunataks (Fig. 2c), and sand for yardangs (Fig. 2d).

Comparison of paleo-valley patterns with terrestrial stream valley types suggests that fluvial erosion may have affected the old tesserae terrain in support of models for more Earth-like conditions in Venus’s earlier history. This hypothesis can be fully tested with higher-resolution data from future Venus missions.

## Results

### Combined tectonics and fluvial erosion

**Fig. 3:**
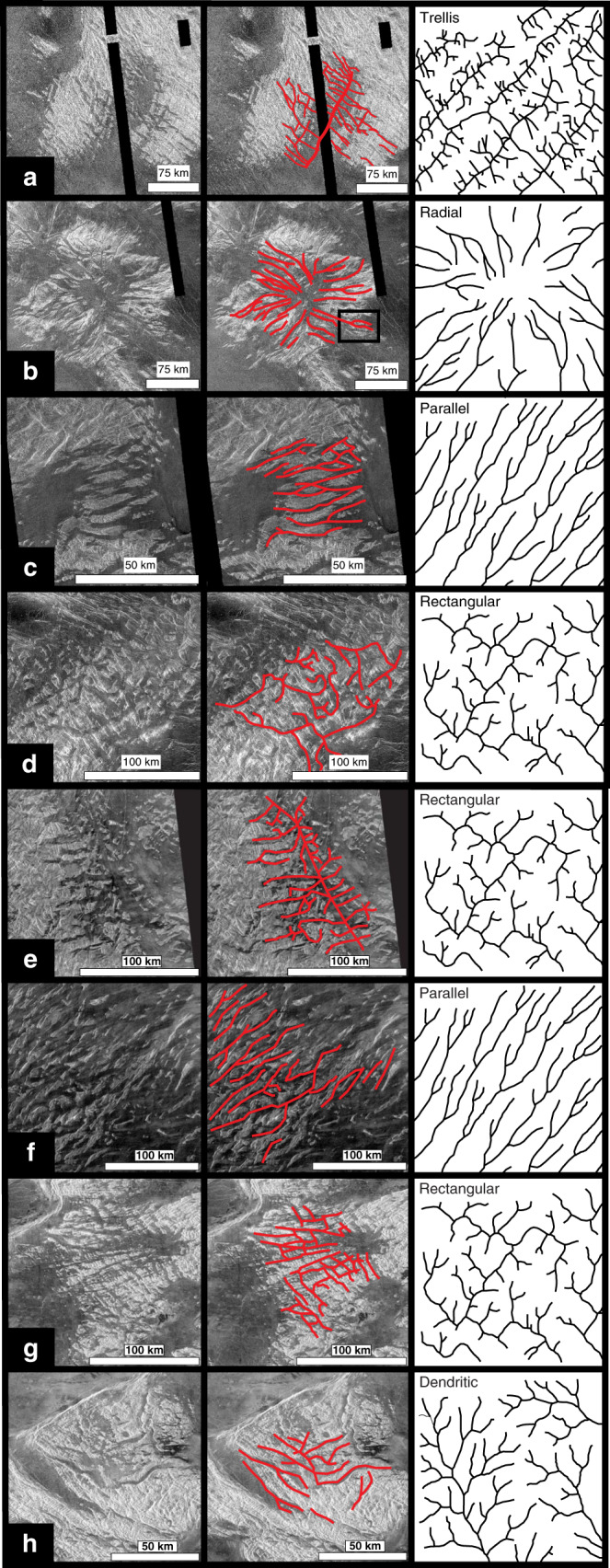
Valley patterns revealed by lava flooding and our fluvial interpretation. Shows tesserae regions that are partially flooded by lava flows and our interpretation of the resultant patterns in terms of stream drainage patterns. Each row features a Magellan SAR image (left) on which an interpreted valley pattern is drawn based on the volcanic flooding (centre) and which is matched to terrestrial stream drainage patterns (right). Types of stream drainage on Earth are after Howard (1967)^35^ and Bridge and Demicco (2008)^36^. Centre location of SAR images: rows **a** (4.4°S, 48.2°E), **b** (16.3°S, 57.3°E), **c** (4.6°S, 62.1°E), **d** (7.5°S, 68.8°E), **e** (4.6° S, 74.4° E), **f** (1.1° S, 69.8° E), **g** (50.8° N, 129.0° E), and **h** (48.9° N, 126.4° E). The SAR images are left-looking Cycle 1 from the Magellan mission; NNW–SSE-trending black bands are gaps in the radar image data. North is to the top in each image. The red box in row b shows the location of Figs. 4a and 5. White lines were drawn through all major valleys (see text for limitations of this proxy approach).

The eight tesserae regions of this study (Fig. 3)^33^ are located in Venusian quadrangles V-33, V-34, V-12, and V-4. Quadrangles V-33, V-34, and V-12 are currently unmapped and the northernmost portion of Fig. 3g, h are in mapped quadrangle V-4^34^. Given their tectonic complexity, the geological mapping of tesserae (including in Quadrangle V-4) has naturally focussed on structural mapping (such as folds and faults) with limited lithological mapping (apart from distinguishing areas of intra-tesserae plains units); published maps have mostly been shaped by the standard implicit assumption in the literature (e.g., refs. ^4–6,27^) that tesserae did not experience erosion. This viewpoint requires all changes in surface topography to solely reflect tectonic processes, such as folding and faulting (Fig. 1b). From a purely tectonic perspective, the tessera paleo-valley pattern in Fig. 1 should be interpreted as a broad synclinal flexural fold with a wavelength of 120 km flooded by lava and flanked by anticlines in the tesserae on east and west sides (Fig. 1b). An alternative but again purely tectonic interpretation would be a fault-bound rift with a width of 30–50 km. However, the volcanic-flooding pattern (Fig. 1e) does not delineate a simple down-dropped crustal block (a rift), and there is no obvious continuation of rift structures outside the area of volcanic flooding to the NNE (north-northeast); such features should be obvious in the absence of erosion. Two other possible geological interpretations are pure erosion of layered stratigraphy (Fig. 1c) and a combination of synclinal folding followed by erosion (Fig. 1d). The basin-parallel lineament sets in the tessera hills on the east and west flanks of the valley appear to belong to faintly visible surfaces that are inward dipping toward the synclinal axis (Fig. 1e; see white dashed lines) and appear to be inconsistent with either pure tectonic deformation (Fig. 1b) or pure erosion (Fig. 1c) models. The simpler interpretation (acknowledging the limits of image and topographic resolution) is development of synclinal fold plunging to the south (direction of arrow) followed by extensive fluvial erosion (Fig. 1a) to produce a widened valley and topographically subdued small tesserae inliers within that valley. It is noteworthy that given the 20× vertical exaggeration in Fig. 1e, which the steeply dipping surfaces implied by the lineaments, would correspond to significantly shallower true dips; for instance, an apparent 80° dip in Fig. 1e would correspond to a true dip of 15°.

**Fig. 4:**
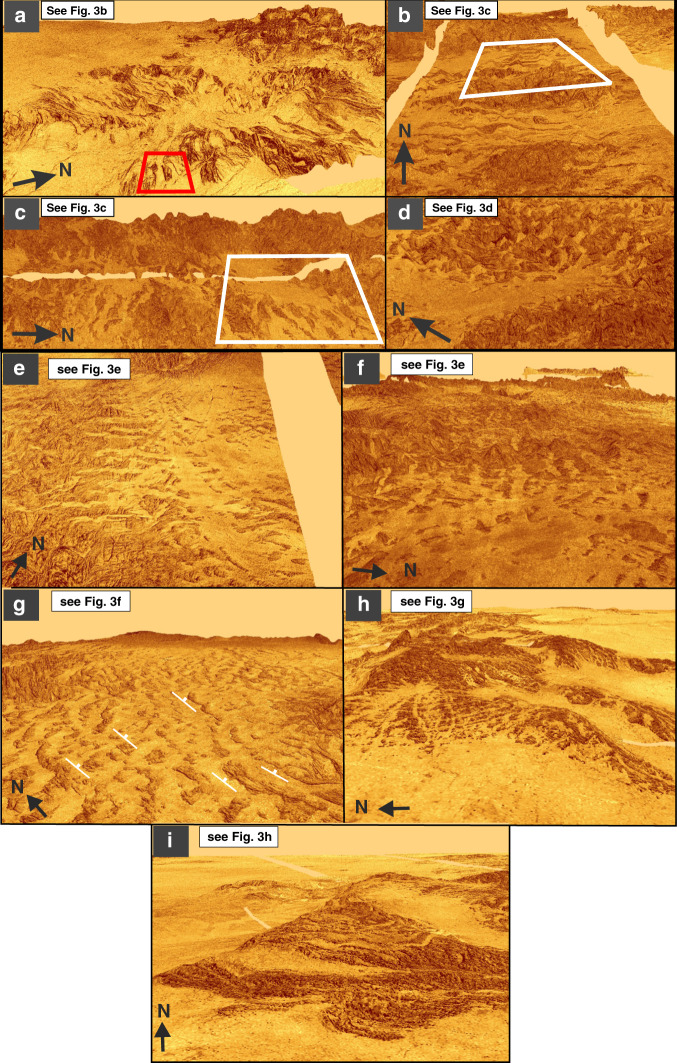
Elevated oblique 3-D views of tesserae flooded by mafic lavas. Images were generated from SAR images draped on the Magellan altimetry topography using the software ArcScene (vertical exaggeration 20×). These images correspond to the areas shown in Fig. 3: **a** corresponds to area of Fig. 3b viewed from east (red box explained in Figs. 3b and 5 and discussed in text); **b** Fig. 3c viewed from south (white box gives approximate location of Fig. 3c); **c** Fig. 3c viewed from east (white box gives approximate location of Fig. 3c); **d** Fig. 3d viewed from south-southwest; **e** Fig. 3e viewed from south-southeast; **f** Fig. 3f viewed from east; **g** Fig. 3f viewed from southwest; white strike-and-dip symbols denote potential geological bedding (flood basalt sequence?) dipping to the east; **h** Fig. 3g viewed from west, and **i** Fig. 3h viewed from south. White boxes in **b** and **c** give approximate location of Fig. 3c. All surfaces viewed from an elevation angle of about 20°. False horizon at top of images due to limited extent of data clipped for processing in ArcScene. Additional versions of **b**–**g** were derived through draping the SAR image on the stereo-topography of Herrick et al.^31^ and are available in Supplementary Information. The images in Supplementary Fig. 1 do not appear to provide any better resolution than corresponding ones in Fig. 4.

Building on these observations, additional areas of tesserae are considered for evidence of fluvial erosion (Figs. 3 and 4). SAR images of selected areas (Fig. 3) allow paleo-valley patterns to be mapped and compared with types of stream drainage patterns on Earth^35,36^ (left sketch). Elevated oblique three-dimensional views (Figs. 1e and 4) of each of these regions in Fig. 3 were generated from SAR images draped on the Magellan altimetry topography; these provide visual support for the interpretation that younger lava flows mark paleo-valleys in tesserae margins. Additional oblique views were generated (Supplementary Fig. 1) by draping the SAR images on the stereo-topography data^31^. Lava flooding will preferentially fill major valleys near the tessera margins and will less reliably flood the valley networks further inland and upslope within the tesserae.

**Fig. 5:**
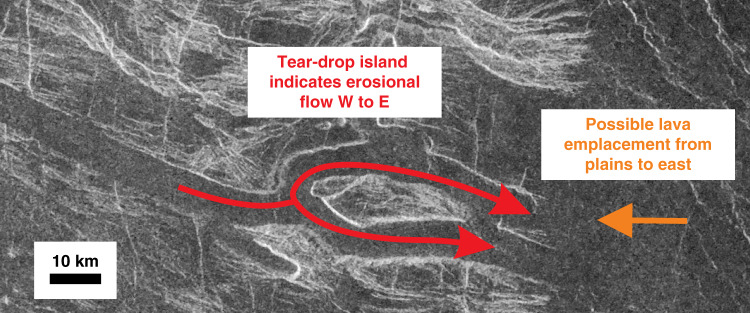
Tear-drop shaped island. A high-standing portion of a tessera unit that resembles a tear-drop-shaped island. Centre of image is at 16.7°S, 058.0°E. North is to the top. For location in a broader context, see red box in Figs. 3b and 4a.

While recognizing this limitation of the proxy approach, it is notable that the mapped tessera valley patterns in Figs. 3 and 4 are morphologically similar to five of the six recognized terrestrial stream drainage patterns^35,36^, which include trellis, radial, parallel, rectangular, and more speculatively dendritic (leaving only the annular pattern unrecognized). The morphological and scale similarities between these candidate tessera paleo-valley patterns and stream drainage patterns on Earth are consistent with a scenario under which fluvial erosion contributed to shaping tessera topography—which likely had already been affected to some degree by earlier folding and faulting tectonics. A further example of interpreted fluvial erosion is offered in Fig. 5, where a kipuka that we interpret as a tear-drop-shaped island is present in a lava-flooded valley within a tessera. The tear-drop geometry suggests fluid flow from west to east.

Terrestrial stream drainage patterns are controlled by rock properties and slopes^35–37^ and thus the type of stream drainage pattern that best matches the flooded geometries within a particular tessera could be indicative of control by local geology. As a generalization, radial, parallel, and dendritic patterns are formed on slopes underlain by homogenous material, although parallel patterns can also reflect steep layered sequences with resistant and weak beds alternating. Trellis and rectangular patterns can be caused by dipping sedimentary strata of unequal resistance to erosion, by dipping volcanic flows with differential erosion between the upper and lower portions of flow, and/or by orthogonal pre-existing fracture patterns. Radial patterns develop on topographic domes or basins composed of homogenous material.

For each example in Fig. 3, we consider both a tectonic and fluvial erosion interpretation, and favour a combination of initial tectonics followed by erosion. As mentioned above, the pattern in Fig. 3a (cf. Fig. 1) is consistent with a NNE–SSW (north-northeast - south-southwest)-trending plunging syncline (shown by the direction of arrow) that has been widened by erosion. The edge of a layered (possibly volcano-sedimentary?) sequence is exposed on the eastern and western flanks of the basin (apparently steep but given the vertical exaggeration the true dip may only be 10 s of degrees). The radiating pattern in Fig. 3b (see Fig. 4a) has previously been inferred to represent a valley network caused by lava flows (Fig. 4 in ref. ^18^) and this interpretation could be consistent with subsequent lava flows filling older valleys that had been generated by fluvial erosion. However, in detail this pattern is complicated and is consistent with radial drainage in some places, but not in others. One explanation is that the there was significant tectonic modification after the creation of the radial drainage system but before lava emplacement.

The pattern in Fig. 3c (see also Fig. 4b, c) could be interpreted as a series of parallel ENE (east-northeast)-trending graben or as an anticlinal–synclinal sequence with a fold spacing of 4–10 km followed by erosion. The flooded tessera pattern in Fig. 3d has some resemblance to orthogonal jointing in uplifted plateaus of uniform rock, or to chocolate tablet boudinage structures^38^ subsequently exhumed by erosion. We interpret the flooded pattern in Fig. 3e (as well as Fig. 4e, f) as having been controlled by two tectonic trends: a NNW–SSE-trending rift and a set of perpendicular, ENE–WSW (east-northeast - west-southwest)-trending geological lineaments, with all features widened by subsequent erosion. Figure 3f (see also Fig. 4g) can be interpreted as layered stratigraphy dipping to the ENE and exposed by erosion (cf. ref. ^39^). Figure 3g (see also Fig. 4h) can be interpreted as planar surfaces associated with two sets of orthogonal lineament sets of uncertain origin (northwest- and northeast-trending) that have, again, been widened by erosion. Figure 3h (see also Fig. 4i) shows complicated primary geology with multiple trends of curving and straight lineaments of uncertain origin and uncertain dip widened by erosion and later filled by lava. The variable topography suggests subsequent uplift, but in this case the lava flows would preserve the paleo-valley pattern that was present when those lava flows were emplaced.

It is important to note that the width of lava flooding in the proposed tesserae valleys (up to >50 km) should not be interpreted as equalling the width of the postulated river systems. Depending on their accumulated thickness, the infilling lava flows could fill the valleys to a level that obscures the details of the past fluvial system beneath and prevents quantitative morphological analysis of the valley patterns, which could include, meandering rivers, mega-floods, and even glaciation. Terrestrial river systems (and similar water and methane valley systems on Mars and Titan, respectively) can be described quantitatively in terms of width, length, changes in orientation, sinuosity, spacing, and degree of interconnectivity^37,40–42^. Although beyond the scope of this initial study, such a sophisticated morphometric analysis using higher-resolution topographic data from future Venus missions will allow robust testing of our hypothesis that the features we report are analogues to fluvial systems on Earth, Mars, and Titan.

### Wind and glacial erosion

There are two classes of aeolian features that occur in volcanic areas of Venus: depositional and erosional. Depositional features include radar dark parabolic halos, wind streaks, and dunes and ergs (dune fields at least 125 km^2^ in area^43^). Parabolic patterns of wind-transported and -deposited ejecta material occur downwind from impact craters^44^. At least two ergs have been recognized in the Magellan SAR images: the Aglaonice (unofficial name; 25°S, 340°E; covers ~1290 km^2^) and the Meshkenet (unofficial name; 67°N, 90°E; covers ~17,120 km^2^) dune fields^45^. Of the types of aeolian erosional features observed on Earth (e.g., yardangs, zeugen, rock pedestals, and deflation basins), only yardangs have so far been observed on Venus. One example of a yarding field is concentrated in an area 300 km southeast of Mead crater and averages 25 km long and 0.5 km wide^45^. Yardangs and zeugens can be distinguished from wind streaks by having well-defined boundaries and not being situated downwind from topographic highs. Although yardangs have been reported in the post-tessera volcanic regions, the important question in the context of the present study is whether such wind erosion could explain the topographic variations in tesserae. In terms of appearance, both yardangs and zeugens tend to be parallel and elongated (Fig. 2d); such wind-eroded landforms could account for only those paleo-valleys (and the ridges between them) that are linear (Fig. 3c, f and Fig. 4b, c, g). The other types of valley patterns in Figs. 3 and 4 do not exhibit parallel valleys and ridges, and thus are unlikely to have been shaped by wind.

For completeness, we also consider the erosive potential of glaciation, a widespread phenomenon on Earth, not only in the Pleistocene but also at various times in the Phanerozoic, and during earlier Snowball Earth glaciations in the Neoproterozoic and Paleoproterozoic^46,47^. Glacial features have not been observed on Venus and obviously cannot occur under current surface temperatures. However, as noted previously^23^, climatic conditions prior to the runaway greenhouse event may have been favourable for liquid water. Within the uncertainties of these climate models, it is possible that snow and ice might once have been present at high latitudes and elevations within the units that later became tesserae (e.g., in Simulation 28 in ref. ^24^). On Earth, a wide range of glacial landforms exist, including cirques, aretes, horntarn, paternoster lakes, hanging valleys, U-shaped valleys, ribbon lakes, and fjords^48,49^. Most of these morphological features, however, would only be recognized in the higher elevation areas, on the sides of paleo-valleys, for which we have poor altimetric resolution in the Magellan images. In addition, in a dramatically warming environment (during the early stages of Venus’ runaway greenhouse-warming event), any evidence of glacial formation might have been totally overprinted by water erosion.

## Discussion

The suggestion of fluvial erosion in tesserae provides a new interpretative framework for this important but enigmatic terrain type on Venus; four implications of this interpretation are discussed (linking stream drainage pattern with underlying geology, interpretation of lineament patterns in tesserae, interpreting some intra-tessera plains units as erosional remnants of flows, and resolving the age conundrum of tesserae).

As noted above, terrestrial stream drainage patterns are controlled by rock properties and slopes, and thus the type of stream drainage recognized through interpreted paleo-valleys in tesserae could be indicative of local geological structures. In this study, we proffer geological interpretations gleaned from paleo-valley patterns in eight tesserae regions. More widespread application of this technique could contribute to regional reconnaissance-level geological interpretation for partially lava-flooded tesserae across Venus.

Tesserae are characterized by complex sets of lineaments, which can be irregular and/or curvilinear, and which have been interpreted as the surface expression of multiple generations of faults, grabens, and fold crests^3–6^. A fluvial erosion model expands the range of possible interpretations of tesserae lineaments to also include volcano-sedimentary stratigraphic packages (cf. Fig. 3g), flood basalts or layered intrusions that have been differentially eroded^39^.

Within tesserae, there are smooth, intra-tessera plain units interpreted to be isolated volcanic flows^6^ or potential sedimentary accumulations^39^. These intra-tessera units of volcanic origin can be spatially distinct from the global-scale volcanic flooding that embay the tesserae. Although some intra-tessera plains lack recognized volcanic sources and are not of sedimentary origin, they could be erosional remnants of more widespread volcanic events (flood basalts) occurring in tesserae time, prior to the main period of volcanic resurfacing.

Based on impact crater size–frequency distributions, tesserae have an age only slightly older than the widespread smooth plains volcanism^27^, even though the former are much more geologically complex. However, tesserae could be even older if fluvial erosion and other processes were continuously removing traces of impact craters, consistent with observations of deformed or partially flooded craters in tesserae features^50,51^. Once runaway greenhouse warming commenced, fluvial erosion would have stopped and cratering would begin to be better preserved, giving tesserae apparent ages comparable to those of the widespread volcanic units. Thus, the recognition of erosion of tessera could indicate that these enigmatic surface units have significantly more protracted geological histories than previously recognized, even back several billion years.

## Methods

### Technique using partial flooding to recognize paleo-valleys

Figure 2 shows the utility of a younger fluid medium that floods pre-existing topography to visually reveal the location of paleo-valleys. Flooding of tesserae by younger lavas is a critical tool in this study, for identifying paleo-valleys for comparison with terrestrial stream patterns.

### Oblique images

Elevated oblique views of the Venusian surface were generated using the ArcScene feature of ArcGIS by overlaying full-resolution SAR images on the Magellan altimetry data (Fig. 4), which has a 10–20 km horizontal resolution and 50–100 m vertical resolution, and on the stereo-topography data of Herrick et al.^31^, which has an improved horizontal resolution of 1–2 km (Supplementary Fig. 1). Each of the resulting ArcScene images in Fig. 4 and Supplementary Fig. 1 were oriented to produce the most informative views of the topographic relationship between tesserae and embayment by younger lava flooding.

## Supplementary information

Supplementary Information

## Data Availability

Source Magellan SAR images and topographic data are available from the USGS (https://astrocloud.wr.usgs.gov) and the stereo-topography from Herrick et al.^31^.
