# Supplementary material for: Tesserae on Venus may preserve evidence of fluvial erosion
Source: Nat Commun. 2020 Nov 13;11:5789. doi: 10.1038/s41467-020-19336-1 (PMC7666114; doi:10.1038/s41467-020-19336-1)
Supplement: Supplementary file 1 — Supplementary Information [file 41467_2020_19336_MOESM1_ESM.pdf]

Supplementary Note 1

Tesserae on Venus may preserve evidence of fluvial erosion

S. Khawja[1], R. E. Ernst[1,2*], C. Samson[1], P. K. Byrne[3], R.C. Ghail[4] and L. MacLellan[1]

[1]*Department of Earth Sciences, Carleton University, 1125 Colonel By Drive, Ottawa, Ontario K1S 5B6, Canada*

[2]*Faculty of Geology and Geography, Tomsk State University, 36 Lenin Avenue, Tomsk 634050, Russia*

[3]*Department of Marine, Earth, and Atmospheric Sciences, North Carolina State University, Raleigh, NC 27695, USA*

[4]*Department of Earth Sciences, Royal Holloway University of London, Egham, TW20 0EX, United Kingdom*

*Corresponding author.

E-mail addresses:

first author: sarakhawja@cmail.carleton.ca (S. Khawja).

corresponding author: richard.ernst@ernstgeosciences.com (R.E. Ernst).

This section shows and briefly discusses oblique view 3-D diagrams generated in ArcScene using the stereo-topography of Herrick et al.[1] for comparison with similar ArcScene diagrams in Figure 4 which use the Magellan altimetry data.

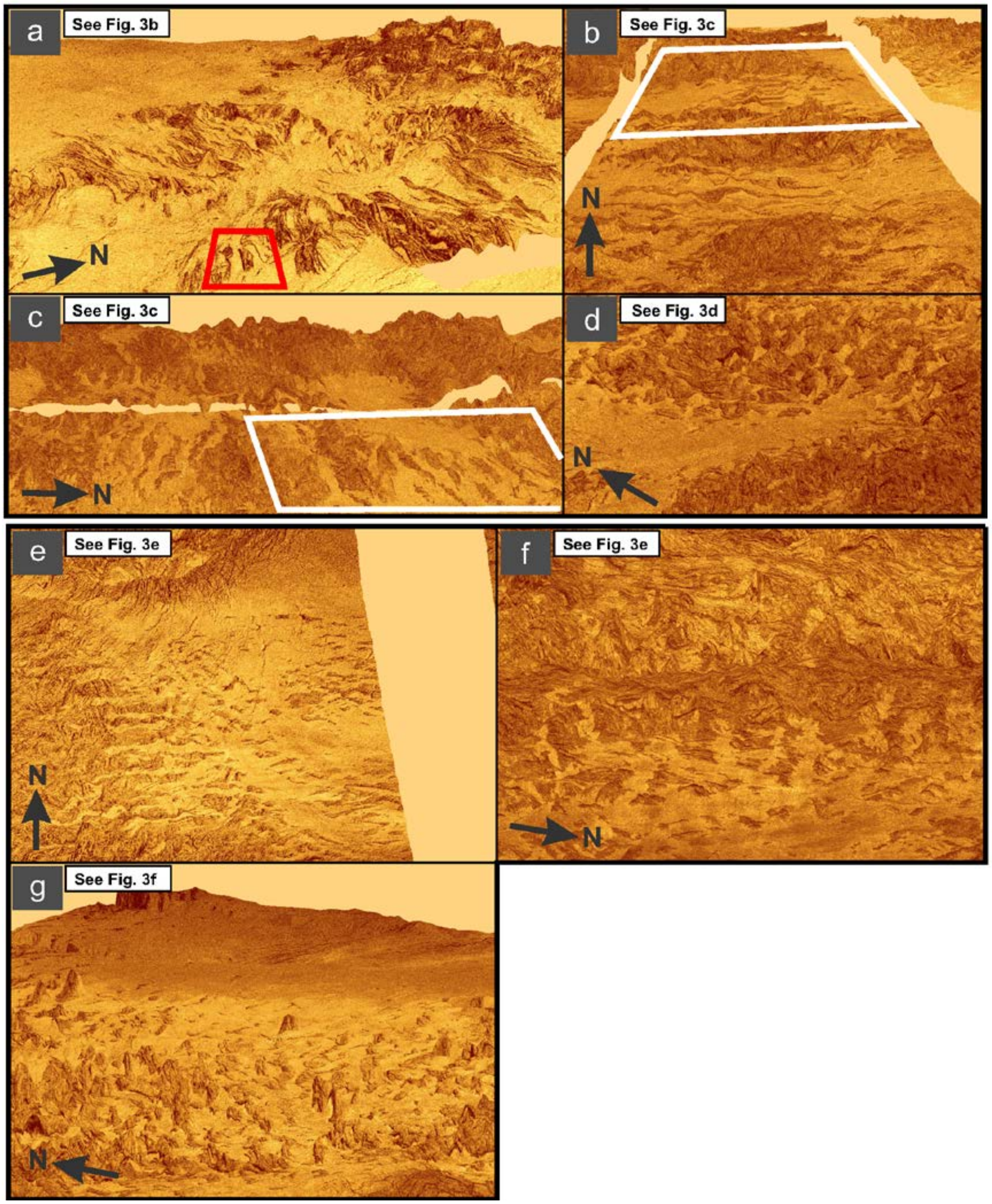

Supplementary Figure 1. Elevated oblique 3-D views of selected tesserae regions which have been flooded by mafic lavas. . Images were generated from SAR images draped on the stereo topography[1] using the software ArcScene (vertical exaggeration 20X). These images are comparable to Fig. 4 in the main text where the SAR images were draped on Magellen altimeter topography.

Note that the spatial resolution of the stereo-derived topography[1] is approximately a factor of ten better than the Magellan altimeter in rough terrain and so in principle should provide better results. However, stereo data rely on matching points with good contrast in both the standard (Cycle 1) and the stereo (Cycle 3) images. Unfortunately, the combination of incidence angle and degradation of the radar instrument and tape recorder can result in poor contrast and unreliable matching points. The additional problem of orbit (ephemeris) errors mean that in practice we find that the ArcScene images generated with stereo-derived topography (Supplementary Figure 1) do not offer a significant improvement over the ArcScene images generated with the Magellan altimeter data (Fig. 4).

Supplementary Reference

1 Herrick, R.R., Stahlke, D.L. & Sharpton, V.L. Fine-Scale Venusian Topography from Magellan Stereo Data, *Eos* 93 (12), 20 (2012).
